# Supplementary material for: Complex‐centric proteome profiling by SEC‐SWATH‐MS
Source: Mol Syst Biol. 2019 Jan 14;15(1):e8438. doi: 10.15252/msb.20188438 (PMC6346213; doi:10.15252/msb.20188438)
Supplement: Supplementary file 8 — Dataset EV7 [file MSB-15-e8438-s008.zip › feature_plots_string/Q14997.pdf]

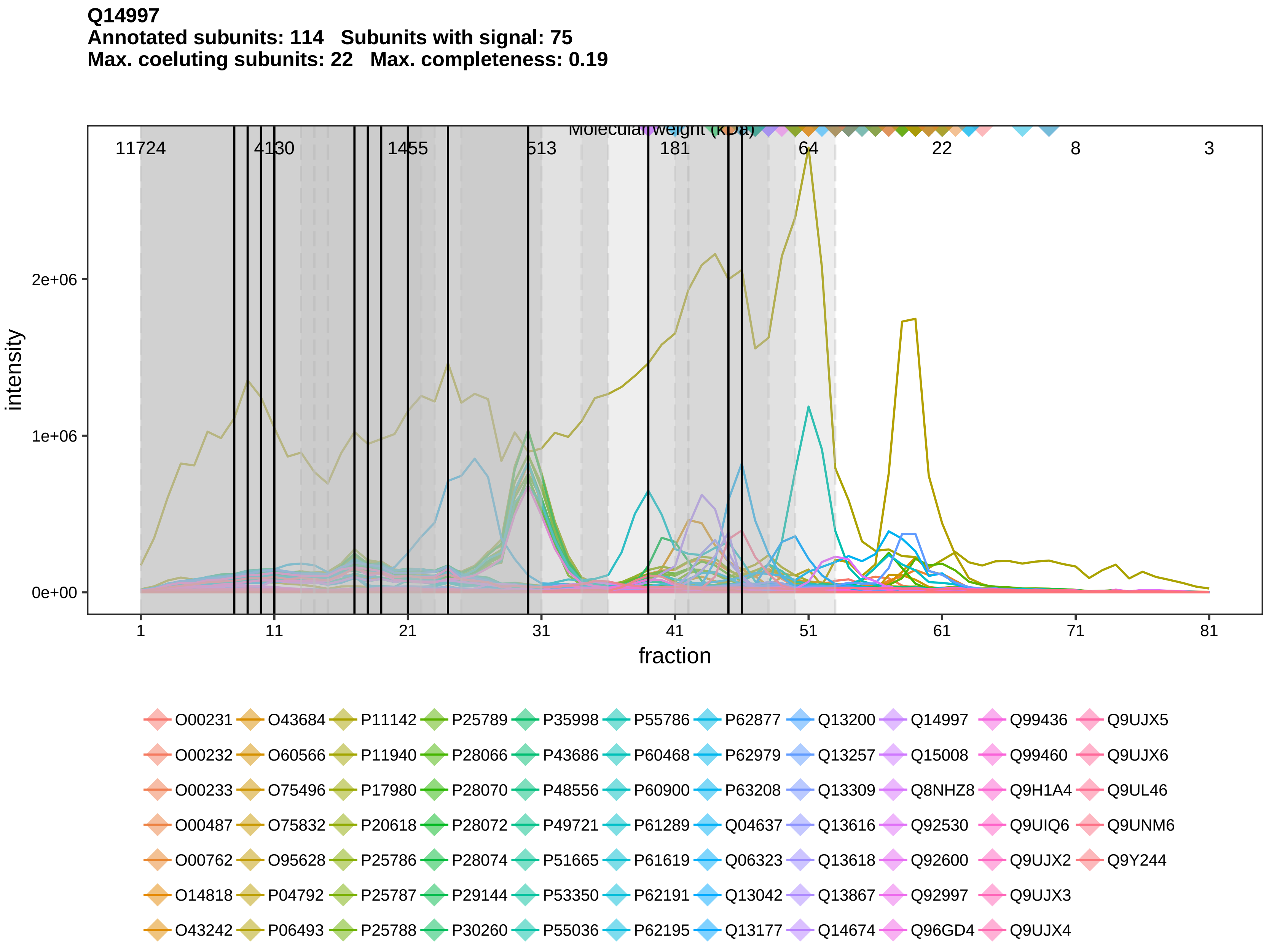
Q14997
Annotated subunits: 114 Subunits with signal: 75
Max. coeluting subunits: 22 Max. completeness: 0.19
Molecular weight (kDa)
11724
4130
1455
513
181
64
22
8
3
intensity
2e+06
1e+06
0e+00
1
11
21
31
41
51
61
71
81
fraction
O00231 O00232 O00233 O00487 O00762 O14818 O43242
O43684 O60566 O75496 O75832 O95628 P04792 P06493
P11142 P11940 P17980 P20618 P25786 P25787 P25788
P25789 P28066 P28070 P28072 P28074 P29144 P30260
P35998 P43686 P48556 P49721 P51665 P53350 P55036
P55786 P60468 P60900 P61289 P61619 P62191 P62195
P62877 P62979 P63208 Q04637 Q06323 Q13042 Q13177
Q13200 Q13257 Q13309 Q13616 Q13618 Q13867 Q14674
Q14997 Q15008 Q8NHZ8 Q92530 Q92600 Q92997 Q96GD4
Q99436 Q99460 Q9H1A4 Q9UIQ6 Q9UJX2 Q9UJX3 Q9UJX4
Q9UJX5 Q9UJX6 Q9UL46 Q9UNM6 Q9Y244
